# Fixed-Position Quasi-Static Load Calibration and Identification of an Aluminum Wing-Box Test Section Using Surface-Bonded Fiber Bragg Grating Sensors

**DOI:** 10.3390/s26144650

**Published:** 2026-07-22

**Authors:** Zhe Fan, Rui Bao, Junkai Sun, Hao Song

**Affiliations:** 1 National Key Laboratory of Strength and Structural Integrity, Institute of Solid Mechanics, School of Aeronautic Science and Engineering, Beihang University, Beijing 100191, China; by1905067@buaa.edu.cn; 2 AVIC Changcheng Institute of Metrology & Measurement, Beijing 100095, China; sunjk23@buaa.edu.cn (J.S.); sh208@139.com (H.S.)

**Keywords:** aircraft wing-box section, fiber Bragg grating sensor, quasi-static load calibration, temperature compensation, structural health monitoring

## Abstract

**Highlights:**

**What are the main findings?**

- A surface-bonded FBG calibration model identified bending moment, shear force, and torsional moment for the tested aluminum wing-box section under fixed-position quasi-static loading.
- Conditions 7 and 8 were excluded from coefficient estimation and used as held-out cases for fixed-position interpolation-type validation within the prescribed four-point quasi-static loading configuration.

**What are the implications of the main findings?**

- For the tested specimen, boundary condition, sensor layout, and four-point loading positions, the FBG system achieved load-identification errors of the same order as those of resistance strain gauges.
- Generalization to variable loading positions, different boundary conditions, environmental changes, or dynamic loading requires additional experiments and recalibration.

**Abstract:**

Section-load calibration is used in aircraft wing-box testing. This study evaluates a fixed-position quasi-static load-calibration and identification procedure for one 7050 aluminum wing-box test section instrumented with surface-bonded fiber Bragg grating (FBG) sensors. A multi-point FBG network was arranged on the skins and webs using finite-element-guided sensor placement to construct bending-, shear-, and torsion-related response features; strain-free reference FBGs provided temperature compensation. All experiments used the same specimen geometry, fixed-root boundary condition, sensor layout, and four actuator positions. Conditions 1–6 were used for regression calibration, whereas Conditions 7 and 8 were held out for interpolation-type validation within the same loading configuration. The maximum/average relative errors were 6.53%/1.51% for bending moment, 2.62%/0.86% for shear force, and 4.04%/1.23% for torsional moment. These results apply only to local laboratory calibration of the tested configuration and do not establish transfer to other geometries, boundary conditions, sensor layouts, loading positions, environmental conditions, or dynamic loading.

## 1. Introduction

The wing box is the primary load-carrying substructure of an aircraft wing and is responsible for transferring aerodynamic loads, supporting bending moments, resisting shear forces, and maintaining torsional stiffness [1,2]. Accurate evaluation of the load state of a wing-box section is therefore essential for structural design, ground calibration tests, airworthiness assessment, and structural health monitoring. In practical wing tests, the external forces applied to the structure are usually transformed into section loads, such as bending moment, shear force, and torsion, which are then correlated with measured strain responses. Establishing a reliable relationship between local strain measurements and section-level loads is a key step in load calibration and inverse load identification for wing-box structures [3,4,5,6].

Resistance strain gauges are standard instruments for load calibration and are widely used in static, fatigue, and residual-strength tests of airframe structures [7,8]. Controlled laboratory specimens and full-scale aircraft structures have also been used to assess strain-based monitoring methods [9,10]. Dense strain-gauge networks on complex wing-box structures require extensive wiring and may be affected by electromagnetic interference, installation variability, and long transmission paths. These constraints motivate evaluation of multiplexed, electromagnetically immune alternatives for multi-point strain measurement.

Fiber Bragg grating (FBG) sensors combine low mass, electromagnetic immunity, and wavelength-division multiplexing [11,12]. Reported aerospace applications include light-aircraft ground and flight tests [13], structural monitoring [14,15,16,17], and shape measurement of flexible or morphing wings [18,19,20]. Grating fabrication and multi-parameter discrimination methods have also been investigated for coupled thermal and mechanical environments [21,22]. Because an FBG records a local wavelength shift rather than load directly, wing-box load calibration additionally requires temperature compensation, defined sensor locations, and an inverse relation between strain responses and section-load components.

Coupled bending, shear, and torsional deformation produces load-dependent strain on both skins and webs. Skin responses are dominated by bending, whereas web responses contain contributions from shear and torsion; consequently, an individual sensor generally responds to more than one load component. Weak responses reduce measurement sensitivity, and correlated responses increase regression-matrix conditioning. Prior work on multi-parameter FBG sensing and strain-based inverse identification indicates that signal selection and inverse-model construction affect load or deformation estimates [23,24,25]. The sensor locations and section-wise response groups were therefore selected using finite-element strain fields and measured load sensitivity.

The experiment integrates finite-element-guided sensor placement, section-wise strain grouping, section-load calculation, regression calibration, and fixed-position validation for a metallic wing-box test section. Four-point loading generated combined bending, shear, and torsional states. A regression model related the temperature-compensated FBG response features to bending moment, shear force, and torsional moment in three monitored sections. Held-out load combinations were used for interpolation-type validation, and resistance strain gauges provided a benchmark under the same test configuration.

The contribution of this study is the experimental integration and evaluation of finite-element-guided sensor placement, section-wise FBG response features, temperature compensation, regression-based identification, held-out fixed-position interpolation-type validation, and comparison with resistance strain gauges. The evidence is limited to one metallic wing-box specimen with the tested geometry, fixed-root boundary condition, sensor layout, load range, and four-point quasi-static loading positions; it does not establish a general arbitrary-load identification framework.

## 2. Regression-Based Identification of Section Loads from FBG Strain Responses

Section loads were estimated from a calibrated relation between the surface-bonded FBG strain responses and the reference loads applied during calibration. The same formulation was fitted independently for each monitored section.

For each monitored section of the wing-box specimen, the target load components are defined as the bending moment M, shear force Q, and torsional moment T. These three quantities describe the section-level load state generated by the prescribed four-point loading configuration. The reference values of M, Q, and T are calculated from the applied actuator loads and their corresponding moment arms, as described in the loading protocol in Section 3.

For the i-th loading sample or loading condition, the section-load vector is written as

(1) $$y_{i}=[\begin{array}{ccc} M_{\;i} & Q_{\;i} & T_{\;i} \end{array}]$$

where M_i_, Q_i_, and T_i_ are the reference bending moment, shear force, and torsional moment for the *i*-th sample, respectively. Under quasi-static loading and within the elastic deformation range of the aluminum wing-box specimen, the measured strain response can be approximated as a linear function of the applied loads. A linear regression model was fitted to establish the relationship between the FBG strain responses and the section-load components. This linear assumption is valid only for the tested load range, boundary condition, sensor layout, and four-point loading configuration considered in this study.

Before the regression model is constructed, the FBG strain signals are preprocessed by zero-offset correction and temperature compensation. The compensated strain value of the j-th FBG channel under the i-th loading sample is denoted as ε_ij_, where i = 1, 2, …, N, and j = 1, 2, …, *p*. Here, *N* is the number of loading samples used in the regression or validation process, and *p* is the number of selected FBG strain channels used as regression inputs. The physical FBG channels were temperature-compensated, grouped by location and dominant load sensitivity, and combined into representative strain features. The regression input matrix X contains these features rather than every individual channel.

The strain-response vector for the *i*-th sample is defined as

(2) $$\varepsilon_{i}=[\begin{array}{cccc} \varepsilon_{i1} & \varepsilon_{i2} & L & \varepsilon_{ip} \end{array}]$$

To account for possible zero-offsets and installation-related bias, an intercept term is included in the regression model. The input vector is therefore written as

(3) $$X_{i}=[\begin{array}{ccccc} 1 & \varepsilon_{i1} & \varepsilon_{i2} & L & \varepsilon_{ip} \end{array}]$$

For *N* loading samples, the input matrix ***X*** and output load matrix ***Y*** are assembled as

(4) $$X=\left[\begin{array}{c} x_{1}^{T}\\ x_{2}^{T}\\ \vdots \\ x_{N}^{T} \end{array}\right]\in {\mathbb{R}}^{N\times (p+1)},Y=\left[\begin{array}{c} y_{1}^{T}\\ y_{2}^{T}\\ \vdots \\ y_{N}^{T} \end{array}\right]\in {\mathbb{R}}^{N\times 3}$$

The strain–load regression model is expressed in matrix form as follows:

(5) Y=Xβ+E

where $\beta \in {\mathbb{R}}^{(p+1)\times 3}$ is the regression coefficient matrix and $E\in {\mathbb{R}}^{N\times 3}$ is the residual matrix. The three columns of **β** correspond to the regression coefficients for bending moment, shear force, and torsion, respectively. The first row of **β** is associated with the intercept term, while the remaining rows quantify the contribution of each selected FBG strain channel to the identified load components.

The regression coefficients are estimated using the calibration dataset, in which both the FBG strain responses and the corresponding reference section loads are known. The ordinary least-squares solution is obtained by minimizing the squared residual between the reference load matrix Y and the model prediction **Xβ**:

(6) $$\hat{\beta }=\underset{\beta }{\mathrm{argmin}}{\left\|Y-X\beta \right\|}_{F}^{2}$$

where ${\left\|\cdot \right\|}_{F}$ denotes the Frobenius norm. The corresponding normal equation is

(7) $$X^{T}X\hat{\beta }=X^{T}Y$$

If **X** has full column rank and **X^T^ X** is nonsingular, the least-squares solution can be written as

(8) $$\hat{\beta }={\left(X^{T}X\right)}^{-1}X^{T}Y$$

Regression-matrix stability was evaluated before coefficient estimation. Channels with weak load sensitivity reduce the signal-to-noise ratio, whereas correlated channels increase the condition number of the normal matrix and amplify sensitivity to measurement perturbations. Response features were selected according to load sensitivity, repeatability, and independence. Matrix rank and condition number were reported before load prediction.

After $\hat{\beta }$ is obtained, the load components for a new loading sample can be predicted from the corresponding FBG strain input vector **x**_new_ as

(9) $${\hat{y}}_{\mathrm{new}}=X_{\mathrm{new}}\hat{\beta }$$

where ${\hat{y}}_{\mathrm{new}}$ contains the predicted bending moment, shear force, and torsional moment of the monitored section.

The overall calibration and validation procedure is divided into two steps. First, the calibration dataset is used to determine the coefficient matrix $\hat{\beta }$. In this study, the loading conditions used for calibration are not used for validation. Second, the validation dataset is input into the calibrated model to evaluate the predictive capability of the regression relationship.

Let **X**_cal_ and **Y**_cal_ denote the FBG strain matrix and reference load matrix of the calibration dataset, respectively. The regression coefficients are calculated as

(10) $$\hat{\beta }={\left(X_{\mathrm{cal}}^{T}X_{\mathrm{cal}}\right)}^{-1}X_{\mathrm{cal}}^{T}Y_{\mathrm{cal}}$$

or equivalently by a numerically stable least-squares solver. For the validation dataset, the predicted section loads are obtained by

(11) $${\hat{Y}}_{\mathrm{val}}=X_{\mathrm{val}}\hat{\beta }$$

where **X**_val_ is the FBG strain matrix of the validation loading conditions and ${\hat{Y}}_{\mathrm{val}}$ is the predicted load matrix. The predicted loads were compared with the reference loads Y_val_ calculated from the applied loading conditions. Conditions 1–6 were used for calibration, and Conditions 7–8 for validation. Repeated measurements under each loading condition were averaged, and their scatter was used to evaluate repeatability.

Conditions 7 and 8 were excluded from coefficient estimation. Their errors characterize interpolation to held-out load combinations generated with the same specimen, actuator positions, boundary condition, and quasi-static procedure; they do not measure transfer to a different experimental configuration.

Load-identification error was calculated by comparing each predicted load component with its reference value. For load component k, where k denotes M, Q, or T, the absolute error of validation sample i is defined as

(12) $$e_{\mathrm{abs},i,k}=\left|{\hat{y}}_{i,k}-y_{i,k}\right|$$

The relative error is defined as

(13) $$e_{\mathrm{rel},i,k}=\frac{\left|{\hat{y}}_{i,k}-y_{i,k}\right|}{\left|y_{i,k}\right|}\times 100\%$$

where $y_{i,k}$ is the reference value and ${\hat{y}}_{i,k}$ is the predicted value. When the reference value is close to zero, the absolute error should be reported to avoid an artificially large relative error.

For all validation samples, the mean absolute error, root mean square error, and maximum absolute error are calculated as

(14) $${\mathrm{MAE}}_{k}=\frac{1}{N_{\mathrm{val}}}\sum_{i=1}^{N_{\mathrm{val}}}\left|{\hat{y}}_{i,k}-y_{i,k}\right|$$

(15) $${\mathrm{RMSE}}_{k}=\sqrt{\frac{1}{N_{\mathrm{val}}}\sum_{i=1}^{N_{\mathrm{val}}}{\left({\hat{y}}_{i,k}-y_{i,k}\right)}^{2}}$$

(16) $${\mathrm{MaxAE}}_{k}=\max_{i}\left|{\hat{y}}_{i,k}-y_{i,k}\right|$$

The average relative error and maximum relative error can also be calculated to compare the identification accuracy of different load components and different sensor systems:

(17) $${\mathrm{MeanRE}}_{k}\;=\frac{1}{N_{\mathrm{val}}}\sum_{i=1}^{N_{\mathrm{val}}}e_{\mathrm{rel},i,k},{\mathrm{MaxRE}}_{k}\;=\max_{i}e_{\mathrm{rel},i,k}$$

The coefficient of determination R2 evaluates the linearity of the strain–load relationship during calibration:

(18) $$R_{k}^{2}=1-\frac{\sum_{i}{\left(y_{i,k}-{\hat{y}}_{i,k}\right)}^{2}}{\sum_{i}{\left(y_{i,k}-{\bar{y}}_{k}\right)}^{2}}$$

For each load component, the signed validation error is defined as

(19) $$e_{i}={\hat{y}}_{i}-y_{i}$$

where *y*_i_ and ${\hat{y}}_{i}$ are the applied reference value and regression-predicted value, respectively. The bias and its 95% confidence interval are calculated as

(20) $$Bias=\frac{1}{n}\sum_{i=1}^{n}e_{i}$$

(21) $$CI_{95\%}=Bias\pm t_{0.975,n-1}\frac{s_{e}}{\sqrt{n}}$$

where *s*_e_ is the sample standard deviation of the signed errors and *t*_0.975, n−1_ is the corresponding critical value of Student’s t-distribution. Because the three repeated measurements at each load level were averaged before validation, the confidence interval represents the uncertainty of the mean bias across the validation load cases.

These metrics are used in the following sections to assess the calibration linearity, validation accuracy, and comparison between the FBG-based load-identification system and conventional resistance strain gauges.

The regression coefficients are specific to the calibrated specimen geometry, sensor layout, boundary condition, load range, and actuator positions. A change in any of these factors requires recalibration or model updating before load identification.

## 3. Experimental Setup and Data Acquisition for Wing-Box Load Calibration

### 3.1. Wing-Box Specimen and Finite-Element Model

A finite-element model was established before the test to support the selection of strain-sensitive regions and to avoid placing sensors in weakly responsive or highly localized stress-concentration regions. The test article was a 7050 aluminum wing-box section. The material properties used in the finite-element analysis were Young’s modulus *E* = 71 GPa, Poisson’s ratio *ν* = 0.33, and yield strength *σ_y_* = 495 MPa. The spanwise overall length of the specimen was approximately 1332 mm, including a main box length of approximately 1193 mm and an end lug/extension length of approximately 139 mm. The chordwise overall width was approximately 1270 mm, and the effective box width was approximately 1126 mm. The section height was approximately 328 mm, with an inner section height of approximately 180 mm. The main spar region had a thickness of 20 mm, the skin region had a thickness of 1.5 mm, and the root reinforcement region had a thickness of 4 mm. The x-axis was defined along the spanwise direction from the fixed root to the free end, the y-axis was defined along the chordwise/front–rear direction, and the z-axis was defined along the vertical loading direction. The structure was modeled using four-node reduced-integration shell elements (S4R) in Abaqus/Standard. The root section was fully constrained along the fixed edges, and the remaining connecting interfaces were defined as bonded contacts. The root section was fully constrained along the fixed edges to reproduce the experimental fixture condition, and the remaining connected interfaces were defined as bonded contacts to approximate the assembled structural connections under quasi-static loading.

The finite-element model was used to identify strain-sensitive and spatially stable regions for subsequent FBG sensor placement rather than to replace the experimental calibration. A mesh-sensitivity analysis was performed using three mesh levels with global/local element sizes of 40/20, 30/15, and 20/10 mm. The corresponding models contained 15,842/14,976, 27,316/25,988, and 58,204/55,936 nodes/elements, respectively. For a monitored response R, the relative change between two successive mesh levels was calculated as Δ*R* = ∣*R*_k_ − *R*_k+1_∣/∣*R*_k+1_∣ × 100%. A maximum relative change below 2% was adopted as the convergence criterion. From the medium-to-fine mesh, the changes in bending stress, bending strain, torsional stress, and torsional strain were 1.22%, 1.39%, 1.58%, and 1.38%, respectively. The fine mesh was used for the subsequent analysis. The complete mesh-sensitivity results are provided in Table S1 in Supporting Material S1. The actuator loads were applied at the same positions as those used in the four-point loading experiment.

**Figure 1:**
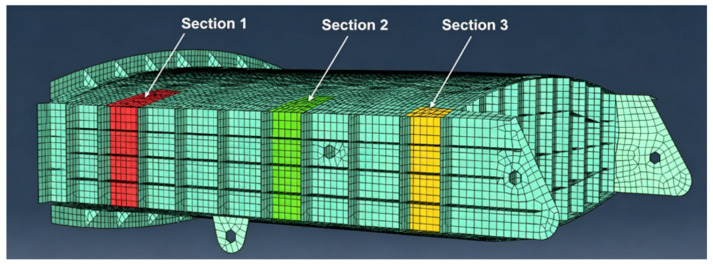
Finite-element shell model of the aircraft wing-box section and monitored-section locations. The monitored sections are highlighted using distinguishable colors and direct labels to indicate their relative spanwise positions.

Table 1 summarizes the main specimen and finite-element modeling parameters. The detailed geometry and section-property definition are shown in Figure 1.

### 3.2. FEA-Guided FBG Sensor Layout and Sensor Grouping

**Figure 2:**
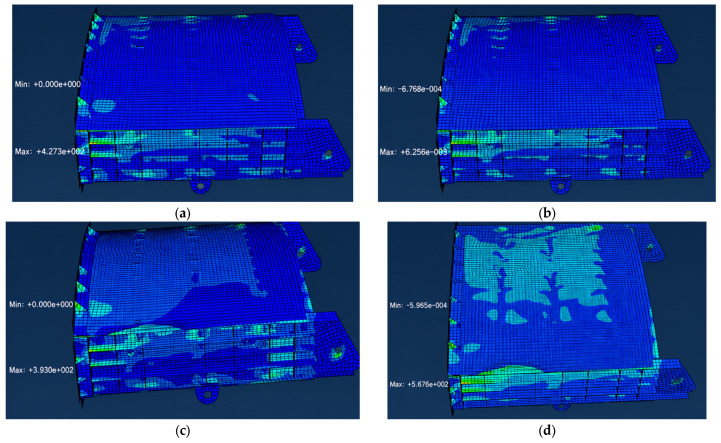
Representative finite-element results used for FBG sensor-layout guidance at the maximum loading level (i = 10): (**a**) von Mises stress distribution under the bending-dominated loading case; (**b**) strain distribution under the bending-dominated loading case; (**c**) von Mises stress distribution under the torsion-dominated loading case; and (**d**) strain distribution under the torsion-dominated loading case.

Representative finite-element results at the maximum loading level (i = 10), used to guide the placement of the FBG sensors, are shown in Figure 2. Specifically, (a) shows the von Mises stress distribution under bending-dominated loading; (b) shows the corresponding strain distribution; (c) shows the von Mises stress distribution under torsion-dominated loading; and (d) shows the corresponding strain distribution.

At the maximum loading level (i = 10), the peak von Mises stress under the bending-dominated condition was 427.1 MPa, with a corresponding peak strain of approximately 6256 με. Under the torsion-dominated condition, the peak stress and strain were 393.0 MPa and approximately 5667 με, respectively. The two peak stresses correspond to approximately 86.3% and 79.4% of the material yield strength of 495 MPa and therefore remained below the yield criterion. These values represent localized finite-element maxima near constrained or geometrically discontinuous regions. The FBG sensors were positioned away from these local stress-concentration regions and were instead installed in regions with sufficiently large but spatially stable strain responses.

Quantitative post-unloading residual-strain statistics were obtained from both the FBG sensors and resistance strain gauges. For the FBG system, the maximum absolute residual strains in Section 1, Section 2 and Section 3 were 3.2, 3.6, and 3.9 με, corresponding to 0.40%, 0.45%, and 0.61% of the respective maximum measured responses. For the resistance strain gauges, the maximum absolute residual strains were 2.9–3.4 με, with residual ratios of 0.37–0.54%. The small residual responses obtained independently from the two sensing systems support a predominantly elastic response at the monitored regions under the prescribed loading conditions. However, complete continuous unloading trajectories were not retained; therefore, a full quantitative evaluation of loading–unloading hysteresis cannot be provided. The complete residual-strain statistics are provided in Table S2 in Supporting Material S1.

The physical FBG channels were organized into sensor groups, from which mathematical strain-response features were constructed without using an electrical-bridge analogy (Table 2). For bending-moment identification, FBG sensors were arranged on the upper and lower skins in three spanwise sections, and the bending-response feature was obtained from the differential strain response between paired upper- and lower-skin sensors. For shear-force and torsional-moment identification, FBG sensors were arranged on the front and rear webs. The averaged web response characterized shear-dominated deformation, whereas the asymmetric front-to-rear web response characterized torsional deformation.

For the (*i*)-th monitored section, the bending-related, shear-related, and torsion-related virtual response features were defined as

(22) $$B_{i}=\frac{1}{3}\sum_{j=1}^{3}\left(\varepsilon_{M,i,j}^{T}-\varepsilon_{M,i,j}^{D}\right)$$

(23) $$C_{i}=\frac{1}{4}\sum_{j=1}^{2}\left(\varepsilon_{ST,i,j}^{B}+\varepsilon_{ST,i,j}^{A}\right)$$

(24) $$D_{i}=\frac{1}{2}\sum_{j=1}^{2}\left(\varepsilon_{ST,i,j}^{B}-\varepsilon_{ST,i,j}^{A}\right)$$

where $\varepsilon_{M,i,j}^{T}$ and $\varepsilon_{M,i,j}^{D}$ are the temperature-compensated strain responses of the upper- and lower-skin FBGs, respectively, and $\varepsilon_{ST,i,j}^{B}$ and $\varepsilon_{ST,i,j}^{A}$ are the temperature-compensated strain responses of the front- and rear-web FBGs, respectively. Tensile strain was defined as positive after temperature compensation. The downward loading direction was defined as positive in the loading system. The sign of each virtual response feature was determined by the sensor position and the above equations, and the corresponding load-response direction was reflected in the fitted regression coefficients.

**Table 2:** Definition of FBG sensor groups and virtual response features.

Sensor Group	Number of FBG Sensors	Bonding Position	Sensing Direction	Gauge Length	Virtual Response Feature	Sign Convention
$\varepsilon_{M,i,j}^{T}$	3 × 3	Upper skin of Section 1, Section 2 and Section 3	Spanwise direction	10 mm	*B_i_*	Tensile strain positive
$\varepsilon_{M,i,j}^{D}$	3 × 3	Lower skin of Section 1, Section 2 and Section 3	Spanwise direction	10 mm	*B_i_*	Tensile strain positive
$\varepsilon_{ST,i,j}^{B}$	3 × 2	Front web of Section 1, Section 2 and Section 3	Along the principal web strain direction	10 mm	*C_i_*, *D_i_*	Tensile strain positive
$\varepsilon_{ST,i,j}^{A}$	3 × 2	Rear web of Section 1, Section 2 and Section 3	Along the principal web strain direction	10 mm	*C_i_*, *D_i_*	Tensile strain positive
Temperature-compensation FBGs	Installed near the strain-measurement regions	Strain-isolated locations	Isolated from mechanical strain as far as possible	10 mm	Temperature compensation	Temperature-induced wavelength shift only

Note: *i* = 1, 2, 3 denotes the monitored section number. For the bending-related sensors, *j* = 1, 2, 3 denotes the point index at each section. For the shear/torsion-related sensors, *j* = 1, 2 denotes the point index at each section. The superscripts *T* and *D* denote upper and lower skins, respectively, while *B* and *A* denote front and rear webs, respectively. The virtual response features *B_i_, C*_i_, and *D_i_* are mathematical combinations of temperature-compensated FBG strain responses and are not electrical bridge circuits.

The bending-moment measurement points were denoted as $M_{i-j}^{T/D}$, where *i* is the section number (*i* = 1, 2, 3), *j* is the point index (*j* = 1, 2, 3), and *T/D* indicates installation on the upper or lower skin, respectively. This arrangement resulted in 18 FBG strain sensors for bending-response measurement. The shear/torsion measurement points were denoted as $ST_{i-j}^{B/A}$, where *i* is the section number (*i* = 1, 2, 3), *j* is the point index (*j* = 1, 2), and B/A indicates installation on the front or rear web, respectively. This arrangement resulted in 12 FBG strain sensors for shear- and torsion-related response measurement. Additional temperature-compensation FBG sensors were installed near the strain-measurement regions and isolated from mechanical strain as far as possible.

**Figure 3:**
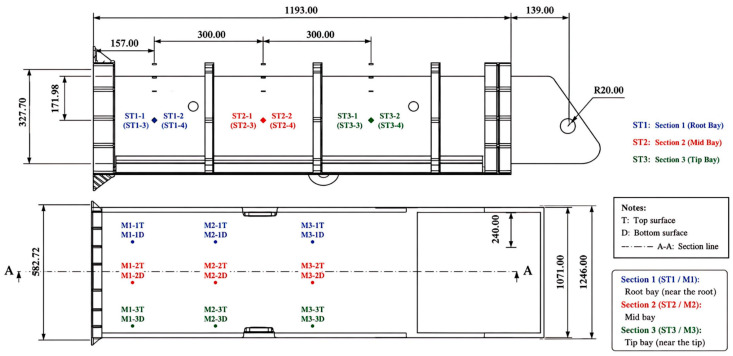
Layout of the surface-bonded FBG sensor groups and monitored sections on the wing-box specimen. Section 1, Section 2 and Section 3 are arranged along the spanwise direction from the fixed root to the free end.

Before the loading test, all surface-bonded FBG sensors were attached to the wing-box specimen according to the bonding and curing procedure. The sensor bonding quality was checked after curing, and the specimen was kept under stable laboratory conditions for 24 h before testing. As shown in Figure 3, three monitored sections were defined along the spanwise direction of the wing-box specimen. Section 1, Section 2, and Section 3 were located at approximately x = 157 mm, 457 mm, and 757 mm from the fixed root, respectively, with an interval of approximately 300 mm between adjacent sections. At each section, bending-related FBG sensors were arranged on the upper and lower skins, while shear- and torsion-related FBG sensors were arranged on the front and rear webs. This layout was designed to capture the main strain features associated with bending deformation, shear-dominated deformation, and torsional asymmetry.

### 3.3. Four-Point Loading Configuration and Section-Load Calculation

**Figure 4:**
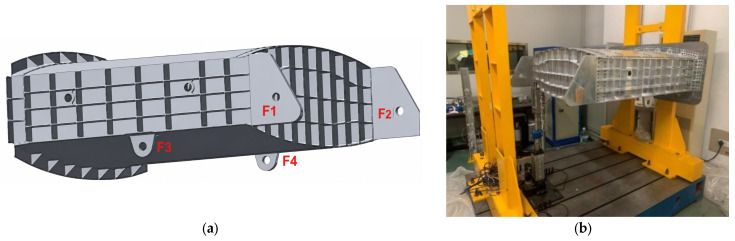
Four-point quasi-static loading experiment on the aluminum wing-box test section: (**a**) actuator loading positions and (**b**) experimental test rig. F1 and F3 act on the front edge, whereas F2 and F4 act on the rear edge.

A four-point loading configuration was used to generate controlled combinations of bending moment, shear force, and torsion in the wing-box section, as shown in Figure 4. The four loading points were denoted as *F_1_*, *F*_2_, *F*_3_, and *F*_4_. Specifically, *F*_1_ was located on the outer front edge, *F*_2_ on the outer rear edge, *F*_3_ on the inner front edge, and *F*_4_ on the inner rear edge of the wing-box section. The downward loading direction was defined as positive. Each loading point was equipped with a hydraulic actuator with a full-scale capacity of 20 kN and an accuracy of ±0.5% full-scale, corresponding to a force accuracy of ±0.10 kN, and spherical hinges were used to reduce loading misalignment.

For a given section *k*, the section-level shear force, bending moment, and torsion were calculated from the applied point loads and their corresponding lever arms. The general calculation can be expressed as

(25) $$Q_{k}=\sum_{x_{m}>x_{k}}I_{mk}F_{m}$$

(26) $$M_{k}=\sum_{x_{m}>x_{k}}I_{mk}F_{m}(x_{m}-x_{k})$$

(27) $$T_{k}=\sum_{x_{m}>x_{k}}I_{mk}F_{m}y_{m}$$

where *Q*_k_, *M*_k_, and *T*_k_ denote the shear force, bending moment, and torsional moment at monitored section k, respectively. *F*_m_ is the downward force applied at loading point m, which is defined as positive. The coordinates x_m_ and x_k_ are the spanwise positions of loading point m and section *k*, respectively, measured from the fixed-root reference plane. Therefore, x_m_−x_k_ represents the spanwise lever arm of *F*_m_ about section *k*. The coordinate y_m_ is the effective transverse moment arm of loading point m with respect to the reference torsion axis. The summation condition x_m_ > x_k_ indicates that only loading points located outboard of section *k* contribute to the internal loads in that section. The spanwise coordinates of Section 1, Section 2 and Section 3 are x_k_ = 157, 457, and 757 mm, respectively. The projected loading-point coordinates (x_m_, y_m_) used in the calculations are F1(635, 1640), F2(635, 500), F3(1322, 1640), and F4(1322, 500) mm. Because the applied forces act vertically, their vertical coordinates are not involved in the calculation of *Q*_k_, *M*_k_, and Tk, as listed in Table 3. Based on these coordinates and the loading schemes listed in Table 4, the calculated internal loads at the three monitored sections are summarized in Table 5.

Conditions 7 and 8 used load-sharing ratios absent from the coefficient-estimation dataset but retained the same fixture and structural configuration as Conditions 1–6.

Thirty resistance strain gauges were installed adjacent to the 30 FBG strain-measurement channels, with matched section, structural region, and measurement direction. Supporting Material S2 lists the strain-gauge labels, corresponding FBG channels, monitored sections, surface locations, measurement directions, and feature groups. Both systems used the same loading protocol and stable-window averaging procedure.

### 3.4. Test Procedure, Data Acquisition, and Preprocessing

The load test was conducted under controlled laboratory conditions. The ambient temperature was maintained at 20 ± 5 °C, and the relative humidity was kept below 85% RH. The FBG sensing system consisted of Luna os1100 single-point FBG strain sensors. The Bragg wavelengths were selected within 1525–1565 nm, and the nominal grating length was 10 mm. The nominal strain sensitivity was approximately 1.2 pm/με. The wavelength signals were recorded using a Luna HYPERION si155 interrogator operated at 100 Hz. Based on the wavelength accuracy of 1 pm and the nominal strain sensitivity, the equivalent strain resolution was approximately 0.83 με. The resistance strain gauges used for comparison were HBK 1-LY13-10/120 gauges with a nominal resistance of 120 Ω and a gauge factor of 2.08. The strain-gauge signals were acquired using an HBK QuantumX MX1615B bridge/DAQ module in a three-wire quarter-bridge configuration at 100 Hz. The FBG and strain-gauge data were recorded using Luna ENLIGHT V1.18.8 and HBK catman AP V1.9.0 software, respectively. The detailed instrument models, serial or certificate identifiers, calibration bases, calibration intervals, acquisition settings, and specified uncertainty limits are provided in Table S3 in Supporting Material S1.

The FBG sensors were bonded with 3M Scotch-Weld DP490 epoxy after abrasion with 600-grit paper and degreasing with acetone. The nominal FBG bondline thickness was (50 ± 10) μm. The adhesive was cured for 10 h at (20 ± 2) °C under contact pressure, followed by 24 h of stabilization. HBK 1-CA80 cyanoacrylate adhesive was used for the resistance strain gauges, with a nominal bondline thickness below 10 μm, following the manufacturer’s instructions. Differences in bonding-layer thickness and installation procedure were included in the interpretation of strain transfer and sensor comparison.

Before loading, each FBG channel was connected to the interrogator, checked at zero load, and assigned an initial Bragg-wavelength reference. The 120 Ω resistance strain gauges had a nominal gauge factor of 2.08 and were sampled at 100 Hz using a bridge conditioner and data-acquisition system. They were installed adjacent to the corresponding FBG regions so that both systems sampled nearby structural responses under identical loads. Exact strain equivalence was not assumed because sensing length, adhesive layer, packaging, and installation differed between sensor types. The comparison therefore concerns load-identification error under the tested configuration rather than sensor equivalence.

Before the formal loading test, the hydraulic actuators and spherical hinges were inspected to ensure that the applied loads were aligned with the intended loading directions. Three preloading–unloading cycles were then conducted using *F*_1_ = 3600 N, *F*_2_ = 3600 N, *F*_3_ = 1200 N, and *F*_4_ = 1200 N. The preloading stage was used to stabilize the loading system, verify sensor bonding quality, and reduce the influence of initial installation effects. After preloading, the specimen was unloaded to the zero-load state, and both the FBG wavelength readings and strain-gauge outputs were re-balanced before the formal test. During data processing, the no-load readings were subtracted to remove the initial offset. Because the test was quasi-static and each load level was held for 30 s, no additional frequency-domain filtering was applied. Instead, abnormal transient points were removed, and the mean value over the stable portion of the holding interval was used for regression modeling and validation.

Each load level was held for 30 s and sampled at 100 Hz, producing 3000 samples per channel. The first 20 s were treated as the loading and stabilization interval and were excluded from averaging. The final 10 s, containing 1000 samples, were defined as the stable window. A sample was retained only when the deviations of all four actuator forces from their respective targets were within ±0.5% of the 20 kN full-scale, equivalent to ±0.10 kN. This absolute criterion was also applicable when the target force of an actuator was zero.

After force-stability screening, a centered 101-point Hampel filter was applied independently to each FBG channel. A point was classified as abnormal when ∣xt − median(x)∣ > 3 × 1.4826 × MAD(x). Abnormal points were removed without interpolation. If more than 5% of the samples in a stable window were rejected, the holding segment was discarded and the test was repeated. Temperature compensation was applied to the retained samples before calculating the stable-window mean. One mean was obtained for each repeated test, and the average of the three repeat means was used as the regression input. Their standard deviation was retained for repeatability evaluation.

During the formal experiment, each loading condition in Table 4 was applied from load level *i* = 0 to *i* = 10. At each target load level, the load was held for 30 s. The FBG wavelength readings, actuator forces, and environmental temperature were recorded during the holding period. For each repeated test, only the mean response over the stable loading-stage window was retained for regression analysis. The standard deviation among the three retained means was used to evaluate loading-stage repeatability. Complete loading and unloading time histories were not retained in the processed dataset. The average value of the stable portion of the holding period was used as the representative strain response for regression modeling and validation. After each loading condition, the specimen was unloaded to the zero-load state, and the residual responses of both the FBG sensors and resistance strain gauges were recorded before proceeding to the next condition. The resulting post-unloading residual-strain statistics are provided in Table S2 in Supporting Material S1.

Temperature compensation was applied to reduce the influence of environmental temperature variations on the FBG strain readings. Six strain-free reference FBG sensors were used, with two reference sensors installed at each monitored section. At Section i, the skin-region reference *R*_Si_ was placed in a loose PTFE sleeve adjacent to the corresponding aluminum skin and was paired with the three upper-skin and three lower-skin measurement FBGs. The web-region reference *R*_Wi_ was similarly installed adjacent to the web region and paired with the two front-web and two rear-web measurement FBGs. The PTFE sleeves maintained local thermal coupling while minimizing mechanical-strain transfer to the reference FBGs. Reference signals from different sections were not averaged. The complete reference-sensor locations and pairing relationships are provided in Table S4 in Supporting Material S1.

This section-specific and region-specific arrangement accounts for spanwise temperature differences and local differences between the skin and web regions. However, smaller gradients within an individual sensor group, such as upper–lower skin or front–rear web differences, were not independently resolved. Their remaining influence was evaluated using the residual-compensation statistics reported in Table S5 in Supporting Material S1.

For each strain-measurement FBG channel, the wavelength shift can be expressed as

(28) $$\frac{\Delta \lambda_{j}}{\lambda_{B,j}}\equiv K_{\varepsilon ,j}+K_{T,j}\Delta T$$

where $\Delta \lambda_{j}$ is the wavelength shift of the *j*-th strain-measurement FBG, $\lambda_{B,j}$ is the initial Bragg wavelength, $K_{\varepsilon ,j}$ is the strain sensitivity coefficient, $K_{T,j}$ is the temperature sensitivity coefficient, $\varepsilon_{j}$ is the mechanical strain, and Δ*T* is the temperature variation. When a nearby temperature-compensation FBG was used as the reference, the compensated mechanical strain was calculated as

(29) $$\varepsilon_{j}^{\mathrm{comp}}=\frac{1}{K_{\varepsilon ,j}}\left(\frac{\Delta \lambda_{j}}{\lambda_{B,j}}-K_{T,j}\Delta T\right)$$

where $\varepsilon_{j}^{\mathrm{comp}}$ is the temperature-compensated FBG strain used in the regression model. The same zeroing and temperature-compensation procedure was applied to all FBG channels before constructing the strain matrix described in Section 2.

Raw wavelength data were processed as follows: (1) subtraction of the no-load wavelength; (2) reference-FBG temperature compensation; (3) removal of transient abnormal points; (4) averaging over the stable holding interval; and (5) averaging of the three repeated measurements, with their standard deviation retained for repeatability analysis. The resulting FBG strain responses were used as regression inputs.

## 4. Results and Discussion

### 4.1. Linearity and Repeatability of FBG Strain Responses

FBG strain data were collected under eight prescribed loading conditions. Conditions 1–6 were used as the calibration dataset to establish the load-identification model, whereas Conditions 7 and 8 were excluded from coefficient estimation and used as held-out cases for fixed-position interpolation-type validation within the same four-point quasi-static loading configuration. For each loading level, the wavelength readings recorded during the 30 s load-holding stage were converted into temperature-compensated strain responses. The mean response over the stable portion of the holding period was used for linear fitting and regression analysis, while the scatter among repeated measurements was used to evaluate the repeatability of the FBG strain responses.

**Figure 5:**
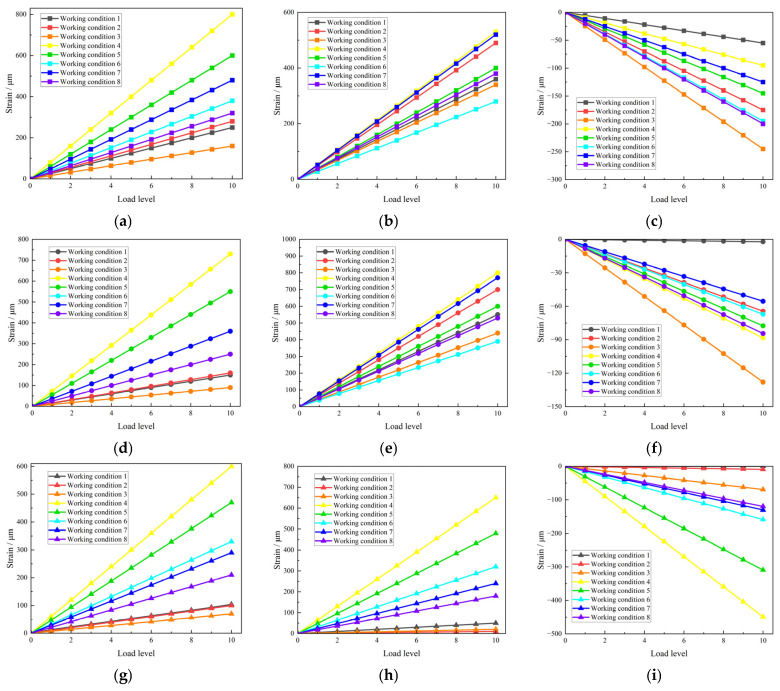
Linear relationship between the averaged FBG response amplitude and loading levels under Calibration Conditions 1–6: (**a**) Section 1 bending response; (**b**) Section 1 shear response; (**c**) Section 1 torsional response; (**d**) Section 2 bending response; (**e**) Section 2 shear response; (**f**) Section 2 torsional response; (**g**) Section 3 bending response; (**h**) Section 3 shear response; and (**i**) Section 3 torsional response.

Figure 5 shows the relationship between the averaged FBG strain-response features and the loading level under Calibration Conditions 1–6. The bending-response features were obtained from the upper- and lower-skin sensor groups, whereas the shear- and torsion-related response features were obtained from the web-mounted sensor groups. For clarity, Figure 5 presents the averaged strain responses and the corresponding linear fitting curves. Full error bars are not shown because each subplot contains multiple calibration conditions, which would reduce the readability of the figure.

Table 6 reports the linear-fit parameters and repeatability statistics. The reported R^2^ ranges quantify linearity over Calibration Conditions 1–6. Across the three repeated loading-stage means, the maximum standard deviations ranged from 0.81 to 4.13 με. These statistics describe the repeatability of stable-window averages and do not measure loading–unloading hysteresis or post-unloading residual strain. The approximately linear responses within the tested range were used for the regression model in Section 2.

At several low load levels, particularly in sections with small strain amplitudes, the scatter increased because of the reduced signal-to-noise ratio. Nevertheless, the strain–load responses remained approximately linear and repeatable within the tested range. The processed FBG response features were therefore used as inputs to the regression model, which should be interpreted only as a local calibration for the tested quasi-static elastic range.

### 4.2. Regression Coefficients and Model Stability

Based on the calibration data obtained from Conditions 1–6, the FBG response-feature matrix and the corresponding section-load matrix were constructed according to the regression formulation described in Section 2. The regression coefficient matrix was then obtained using the least-squares method. The validation conditions were not included in the coefficient-estimation process, so that the model parameters were determined only from the calibration dataset.

The regression uses three mathematical FBG response features, B, C, and D, rather than electrical bridge outputs. Feature B is derived primarily from upper- and lower-skin responses; features C and D are derived from front- and rear-web responses and represent shear-dominated deformation and torsional asymmetry. The three features have different sensitivities to bending moment M, shear force Q, and torsional moment T.

Table 7 lists the regression coefficients of the selected FBG response features. The three columns correspond to bending moment M, shear force Q, and torsional moment T, respectively. Each coefficient represents the contribution of one selected response feature to the corresponding section-load component. The sign of the coefficient reflects the response direction under the defined positive loading direction, while the magnitude reflects the sensitivity of the identified load component to the selected response feature.

**Figure 6:**
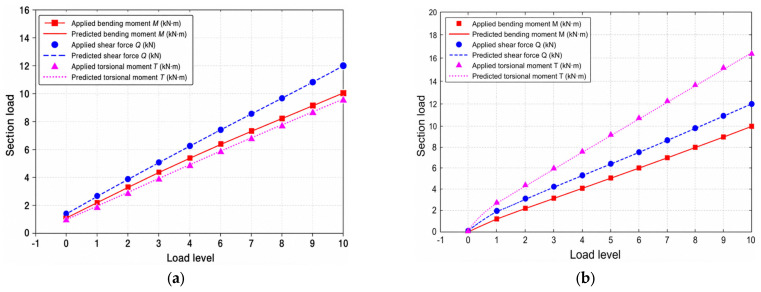
(**a**) Condition 7 and (**b**) Condition 8. Conditions 7 and 8 were excluded from coefficient estimation but used the same loading positions, boundary condition, sensor layout, and quasi-static procedure as the calibration conditions.

Figure 6 shows the overall validation trends, whereas Supporting Material S3 reports every non-zero validation point from Conditions 7 and 8 for all monitored sections. The workbook contains applied and predicted bending moments, shear forces, and torsional moments, together with absolute and relative errors. The maximum and mean relative errors in Table 8 were calculated from these point-wise values.

Although multiple FBG sensors were installed in each monitored section, the regression coefficients in this section were calculated using the constructed virtual strain-response features rather than all individual sensor channels directly. The bending-related feature was constructed from the upper- and lower-skin FBG responses, whereas the shear- and torsion-related features were constructed from the front- and rear-web FBG responses. Thus, each coefficient in the regression table corresponds to one constructed strain feature, not to one physical FBG sensor.

Matrix rank and condition number were used to assess identifiability and collinearity of the response features at each monitored section. Full rank is required to estimate the three load components, whereas the condition number quantifies sensitivity to collinearity. Table 9 reports both quantities.

The response-feature matrix was full rank at each monitored section (Table 9). The condition numbers were 3.63, 2.78, and 19.67 for Section 1, Section 2, and Section 3, respectively. The higher value for Section 3 indicates greater correlation among its response features. All three section matrices were retained for the specified least-squares calibration.

Each coefficient matrix applies only to its calibrated specimen geometry, boundary condition, sensor layout, and loading positions. Changes to these conditions require recalibration.

### 4.3. Fixed-Position Interpolation-Type Validation Under Conditions 7 and 8

Conditions 7 and 8 were not used for coefficient estimation and had front-to-rear load ratios different from those of Conditions 1–6. They nevertheless used the same loading positions, direction, boundary condition, and quasi-static procedure. The reported validation is therefore an interpolation test within the fixed-position load space, not evidence of transfer to arbitrary load positions or dynamic load distributions. No artificial data were added. Evaluation of transfer requires experiments with variable load positions, dynamic loading, or different boundary conditions.

Figure 6 shows the validation curves under Conditions 7 and 8. The comparison between the applied and regression-identified loads indicates that the calibrated model follows the load variation in these held-out cases. The results show that the selected FBG response features retained their load sensitivity for load-sharing combinations not used for coefficient estimation. However, this assessment is limited to fixed-position interpolation-type validation within the prescribed four-point quasi-static loading configuration.

Table 8 reports MAE, RMSE, MaxAE, bias, mean relative error, maximum relative error, and the 95% confidence interval of bias for each load component and monitored section. Each section contributes 20 non-zero points from Conditions 7 and 8; the overall statistics use 60 points. Bending- and torsional-moment errors are given in kN·m, and shear-force errors in kN.

The overall MAE values were 0.0500 kN·m, 0.0461 kN, and 0.0691 kN·m for bending moment, shear force, and torsional moment, respectively. The corresponding RMSE values were 0.0751 kN·m, 0.0587 kN, and 0.0925 kN·m, while the MaxAE values were 0.3537 kN·m, 0.1338 kN, and 0.3120 kN·m. The overall biases were close to zero, namely −0.0005 kN·m for bending moment, +0.0050 kN for shear force, and +0.0033 kN·m for torsional moment. Their 95% confidence intervals all included zero, indicating that no statistically evident systematic overprediction or underprediction was observed within the tested validation dataset.

A leave-one-feature-out analysis quantified the contribution of B, C, and D. For each reduced model, the regression coefficients were refitted with Conditions 1–6 and evaluated with Conditions 7 and 8. Removing B increased bending-moment RMSE from 0.0751 to 0.1877 kN·m and mean relative error from 1.51% to 3.99%. Removing C increased shear-force RMSE from 0.0587 to 0.1469 kN and mean relative error from 0.86% to 2.00%. Removing D increased torsional-moment RMSE from 0.0925 to 0.2774 kN·m and mean relative error from 1.23% to 3.94%. The complete B+C+D model had the lowest RMSE for all three components. Table S6 in Supporting Material S1 reports the full error metrics.

Virtual features were constructed separately for each monitored section; FBG responses were not averaged across sections. Averaging within a sensor group can attenuate an abnormal response from one channel. Each channel was therefore zero-corrected, temperature-compensated, and screened before feature construction, and no channel was rejected by these checks. Because the experiment included no imposed sensor failure or strain-transfer defect, it does not establish fault detection or fault tolerance. Channel-level screening remains necessary before feature construction.

The two validation conditions used fixed actuator positions. The results therefore apply to calibration-based identification for the tested specimen and prescribed four-point quasi-static configuration. Transfer to other loading positions, boundary conditions, environmental states, or dynamic loads was not tested.

### 4.4. Error Characteristics and Comparison with Resistance Strain Gauges

**Figure 7:**
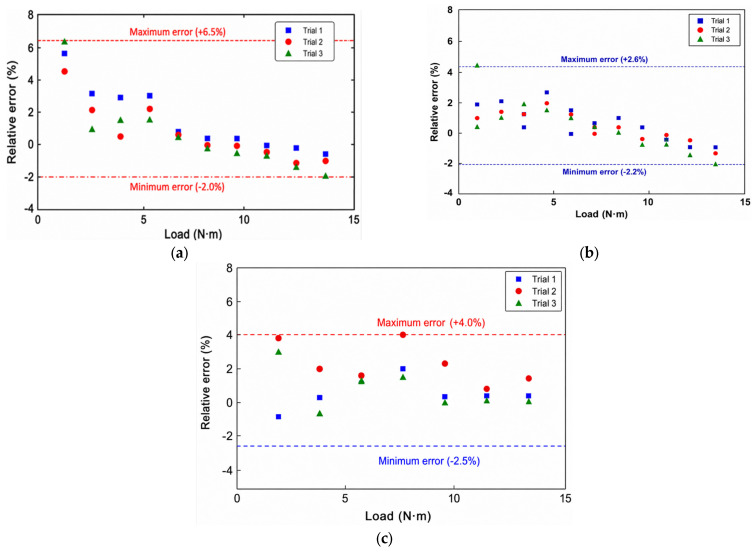
Relative-error distributions of the FBG-based model under the held-out fixed-position validation cases: (**a**) bending moment; (**b**) shear force; and (**c**) torsional moment. The results are limited to Conditions 7 and 8 within the prescribed four-point quasi-static loading configuration. Trial 1, Trial 2, and Trial 3 denote three repeated loading measurements under the same loading condition.

Figure 7 reports the relative-error distributions for bending moment, shear force, and torsional moment under the two held-out conditions. The largest relative errors occurred primarily at low load levels, where small response amplitudes increase the contribution of measurement noise, residual zero offset, and load coupling to the relative-error denominator.

The load-identification accuracy of the FBG-based method was compared with resistance-strain-gauge results using the same calibration–validation split. For the strain-gauge benchmark, Conditions 1–6 were used for regression calibration, whereas Conditions 7 and 8 were excluded from coefficient estimation and used as held-out validation cases. The same linear regression formulation was adopted. For each monitored section, the strain-gauge response features BSG, CSG, and DSG were constructed from the skin and web strain-gauge groups in the same manner as the FBG virtual response features. The regression input matrix was defined as XSG = [1, BSG, CSG, DSG], and the bending moment, shear force, and torsional moment were estimated using ordinary least squares.

The strain-gauge calibration dataset contained 594 repeated records and 198 averaged calibration samples from Conditions 1–6. The validation dataset contained 60 non-zero validation points from Conditions 7 and 8. The strain-gauge layout, regression coefficients, calibration statistics, and complete point-by-point validation results are provided in Supporting Material S2.

For the strain-gauge benchmark, the overall MAE/RMSE/MaxAE values were 0.0498/0.0644/0.1888 kN·m for bending moment, 0.1452/0.1691/0.3435 kN for shear force, and 0.0489/0.0600/0.1535 kN·m for torsion. The corresponding biases were +0.0083 kN·m, +0.0033 kN, and +0.0047 kN·m. The average/maximum relative errors were 1.44%/4.61%, 2.74%/4.07%, and 0.88%/3.09% for bending moment, shear force, and torsion, respectively.

Resistance strain gauges produced lower bending- and torsional-moment errors, whereas the FBG model produced lower shear-force errors. The two systems therefore yielded errors of the same order for the tested quasi-static configuration, with performance dependent on load component.

FBG sensing permits wavelength-division multiplexing, electromagnetic immunity, and low-mass cabling for multi-point measurements. Table 10 also shows that identification error depends on load component, sensor placement, response sensitivity, and regression-matrix conditioning. The results support FBG sensors as an alternative measurement option for the tested wing-box calibration, not as a universal replacement for resistance strain gauges.

An uncertainty budget was established to clarify the main uncertainty sources in the FBG-based load-identification procedure and in the resistance-strain-gauge benchmark. The considered sources include actuator force accuracy, loading-point coordinate uncertainty, interrogator wavelength accuracy, wavelength-to-strain conversion, FBG sensitivity, temperature-compensation residuals, repeated-measurement variability, strain-transfer uncertainty, bondline-thickness uncertainty, gauge-factor uncertainty, and empirical regression residuals. Type-B standard uncertainties were calculated by dividing the specified limits by $\sqrt{3}$ when a rectangular distribution was assumed, whereas Type-A components were evaluated from repeated measurements or regression residuals. For example, the actuator force limit of ±0.10 kN corresponds to a standard uncertainty of 0.0577 kN per actuator. The interrogator wavelength accuracy of ±1 pm corresponds to 0.577 pm, equivalent to 0.481 με using a strain sensitivity of 1.2 pm/με. The repeatability-related standard uncertainty of the mean was 0.47–2.38 με. The empirical regression standard uncertainties were 0.0751 kN·m for bending moment, 0.0587 kN for shear force, and 0.0925 kN·m for torsion. The detailed uncertainty sources, standard uncertainties, and propagation paths are provided in Table S7 in Supporting Material S1.

### 4.5. Temperature Compensation, Repeatability, and Applicability

The measured Bragg-wavelength shift contains mechanical-strain and temperature components. Strain-free reference FBGs adjacent to the measurement regions recorded the local temperature-related shift. This component was subtracted before constructing the regression input matrix.

**Figure 8:**
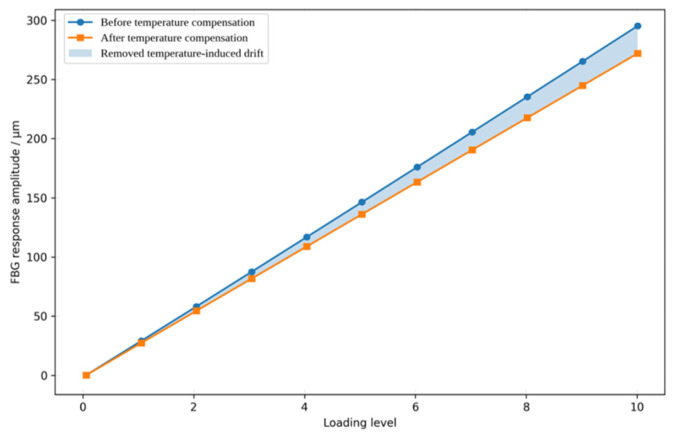
Measured FBG response before and after temperature compensation for the Section 1 bending-related response under Condition 2. The shaded region represents the temperature-induced drift removed from the raw FBG response during compensation.

Figure 8 compares the Section 1 bending-related response before and after temperature compensation under Condition 2. The difference between the curves is the temperature-related component removed during preprocessing; the compensated curve was used to construct the regression feature.

The compensated response shown in Figure 8 was the response feature subsequently used in the strain–load regression analysis. By reducing non-mechanical wavelength drift, the compensation procedure helped improve the stability of the regression input matrix and reduced the influence of environmental temperature variation on load-identification accuracy. To evaluate the temperature-compensation procedure beyond the representative Section 1, Condition 2 example shown in Figure 8, the compensation and preprocessing statistics were calculated for all monitored sections and loading conditions. The analysis included 240 holding segments per section, corresponding to eight loading conditions, ten non-zero load levels, and three repeated tests. The maximum raw thermal components were 18.4, 16.7, and 20.9 με for Section 1, Section 2 and Section 3, respectively. After compensation, the mean absolute residuals were 0.8–1.1 με and the maximum residuals were 2.6–3.4 με. The repeatability standard deviations decreased from 4.9–5.8 με before compensation to 2.4–3.2 με after compensation, corresponding to reductions of approximately 44.8–51.0%. The rejected-point proportions were only 0.21–0.34%. The complete results are provided in Table S5 in Supporting Material S1.

Surface bonding permitted installation, inspection, replacement, and comparison with adjacent resistance strain gauges on the metallic test section. Adhesive degradation, surface damage, and environmental exposure may limit long-term use. Embedded or integrated FBG networks may improve durability, but they require separate calibration and validation under variable load positions, dynamic excitation, environmental variation, and service exposure.

Table 11 cross-references the experimental configuration, regression model, validation metrics, sensor comparison, and associated numerical data.

## 5. Conclusions

This study demonstrated fixed-position quasi-static load calibration and identification for one 7050 aluminum wing-box test section using surface-bonded FBG sensors. Conditions 1–6 were used for calibration, and Conditions 7 and 8 provided held-out interpolation-type validation with the same specimen, fixed-root boundary condition, sensor layout, actuator positions, and quasi-static procedure. Within this limited scope, the maximum/average relative errors were 6.53%/1.51% for bending moment, 2.62%/0.86% for shear force, and 4.04%/1.23% for torsional moment. The FBG and resistance-strain-gauge benchmarks produced errors of the same order, with component-dependent differences; therefore, no universal superiority of either sensing system is claimed. A calibrated load-conditioned strain baseline could support future structural health monitoring studies by separating expected load-related strain variation from changes associated with stiffness loss, local damage, or dynamic response [26,27]. The experiment included no damage states, modal measurements, dynamic excitation, variable loading positions, or service environments and therefore does not validate operational structural health monitoring.

The main contribution is an experimentally evaluated, multi-component section-load calibration procedure for a single metallic wing-box specimen under fixed-position four-point quasi-static loading. The procedure integrates finite-element-guided placement of surface-bonded FBG sensors, section-wise construction of bending-, shear-, and torsion-related response features, temperature compensation, regression calibration, held-out interpolation-type validation, feature-ablation analysis, and comparison with resistance strain gauges. It is neither a new FBG sensing principle nor a general arbitrary-load identification method.

The calibrated coefficients remain specific to the tested specimen geometry, fixed-root boundary condition, FBG layout, load range, and actuator positions. Future work should independently examine other geometries and boundary conditions, embedded or integrated FBG networks, variable loading positions, damage states, dynamic loading, and long-term environmental exposure before the method is considered for operational aircraft load monitoring or structural health monitoring.

## Figures and Tables

**Table 1:** Main specimen and finite-element modeling parameters.

Item	Description
Test article	7050 aluminum wing-box section
Young’s modulus	71 GPa
Poisson’s ratio	0.33
Yield strength	495 MPa
Main spar thickness	20 mm
Skin thickness	1.5 mm
Root reinforcement thickness	4 mm
Element type	Four-node reduced-integration shell element (S4R)
Solver	Abaqus/Standard
Boundary condition	Root section fully fixed along the constrained edges
Interface definition	Bonded contact between connected surfaces
Mesh density	Global shell-element size of approximately 20 mm; local mesh refinement of approximately 10 mm near loading points, constrained regions, ribs, spars, and strain-sensitive regions
Mesh convergence check	Three mesh levels were compared using stress and strain responses. A maximum relative change below 2% between successive mesh levels was adopted as the convergence criterion. Detailed results are provided in Table S1 in Supporting Material S1.
Load application method	The actuator loads were applied at the experimental loading positions F1–F4 as concentrated forces or equivalent nodal forces according to the four-point loading configuration
Coordinate system	The x-axis was defined along the spanwise direction from the fixed root to the free end; the y-axis was defined along the chordwise/front–rear direction; and the z-axis was defined along the vertical loading direction

**Table 3:** Geometric parameters and numerical lever arms used for section-load calculation.

Section	(x_k_)/mm	Load Point	(x_m_)/mm	(y_m_)/mm	(I_mk_)	(x_m_-x_k_)/mm
Section 1	157	F1	635	1640	1	478
Section 1	157	F2	635	500	1	478
Section 1	157	F3	1322	1640	1	1165
Section 1	157	F4	1322	500	1	1165
Section 2	457	F1	635	1640	1	178
Section 2	457	F2	635	500	1	178
Section 2	457	F3	1322	1640	1	865
Section 2	457	F4	1322	500	1	865
Section 3	757	F1	635	1640	0	—
Section 3	757	F2	635	500	0	—
Section 3	757	F3	1322	1640	1	565
Section 3	757	F4	1322	500	1	565

Notes: *I*_mk_ = 1 when *x*_m_ > *x*_k_, and *I*_mk_ = 0 otherwise. The reference torsion axis is *y* = 0. Downward force is positive. The units of F_m_, *x*_m_, and *y*_m_ are N, mm, and mm, respectively.

**Table 4:** Prescribed actuator-load combinations and experimental operating conditions.

Condition	Loading Type	Front Edge		Rear Edge	
		F1 (N)	F3 (N)	F2 (N)	F4 (N)
1	Rear edge only	0	0	1020i	180i
2	Simultaneous front and rear	510i	90i	510i	90i
3	Front edge only	1020i	180i	0	0
4	Inner rear edge only	0	0	0	1200i
5	Inner edges only	0	600i	0	600i
6	Inner front edge only	0	1200i	0	0
7	Simultaneous front and rear	150i	150i	450i	450i
8	Simultaneous front and rear	450i	450i	150i	150i

Notes: 1. *i* represents the load level, *i* = *0*, *1*, *2*…*10*. 2. The downward direction is defined as positive.

**Table 5:** Calculated section loads for the prescribed loading conditions.

Condition		Bending Moment M (kN·m)	Shear Force Q (kN)	Torsional Moment T (kN·m)
Section 1	1	0.69726i	1.2i	0.6i
	2	0.69726i	1.2i	1.284i
	3	0.69726i	1.2i	1.968i
	4	1.398i	1.2i	0.6i
	5	1.398i	1.2i	1.284i
	6	1.398i	1.2i	1.968i
	7	0.9858i	1.2i	0.942i
	8	0.9858i	1.2i	1.626i
Section 2	1	0.33726i	1.2i	0.600i
	2	0.33726i	1.2i	1.284i
	3	0.33726i	1.2i	1.968i
	4	1.03800i	1.2i	0.600i
	5	1.03800i	1.2i	1.284i
	6	1.03800i	1.2i	1.968i
	7	0.62580i	1.2i	0.942i
	8	0.62580i	1.2i	1.626i
Section 3	1	0.10170i	0.18i	0.09i
	2	0.10170i	0.18i	0.1926i
	3	0.10170i	0.18i	0.2952i
	4	0.67800i	1.2i	0.6i
	5	0.67800i	1.2i	1.284i
	6	0.67800i	1.2i	1.968i
	7	0.33900i	0.6i	0.471i
	8	0.33900i	0.6i	0.813i

**Table 6:** Linear fitting parameters and repeatability statistics of FBG strain responses under Calibration Conditions 1–6.

Load Type		Working Condition 1	Working Condition 2	Working Condition 3	Working Condition 4	Working Condition 5	Working Condition 6	R^2^	Max SD/με
Section 1	M	(24.31, −2.77)	(27.25, −1.47)	(15.78, −1.98)	(80.18, −6.30)	(59.11, −4.36)	(37.45, 1.04)	0.992–0.999	4.06
	Q	(35.65, 0.49)	(48.63, 0.44)	(33.68, 0.75)	(52.13, 0.83)	(40.08, 0.48)	(27.69, 0.59)	0.995–0.999	2.40
	T	(−5.16, −3.05)	(−17.51, −1.50)	(−24.75, 0.60)	(−9.75, 0.60)	(−14.41, −1.09)	(−19.39, −0.89)	0.985–0.998	1.31
Section 2	M	(14.48, −1.16)	(15.47, 5.84)	(8.82, 2.62)	(73.03, −6.75)	(54.61, −5.69)	(35.96, −2.02)	0.990–0.999	3.47
	Q	(53.88, 0.94)	(69.53, 0.71)	(44.02, −1.27)	(79.11, 3.96)	(59.77, 0.14)	(38.96, −0.49)	0.992–0.999	4.13
	T	(−0.18, −0.48)	(−6.69, 0.85)	(−12.51, −2.15)	(−8.45, −0.92)	(−7.86, 0.22)	(−6.82, 0.19)	0.982–0.997	0.81
Section 3	M	(10.04, −1.79)	(9.95, −4.66)	(6.71, −5.10)	(60.91, −4.07)	(47.08, −4.77)	(33.06, 0.15)	0.985–0.998	2.54
	Q	(4.47, 2.69)	(0.40, 2.70)	(0.95, 1.12)	(63.86, 4.77)	(47.82, 2.25)	(31.74, 2.18)	0.930–0.995	3.99
	T	(0.59, −6.89)	(−1.01, −1.73)	(−6.95, 3.38)	(−45.00, −1.55)	(−31.00, 0.30)	(−15.96, −0.31)	0.950–0.998	2.48

**Table 7:** Load regression coefficients.

	Bending Moment *M* (kN·m/με)	Shear Force *Q* (kN/με)	Torsional Moment T (kN·m/με)
*B*	0.01956	0.000299	0.00246
*C*	−0.00605	0.02012	−0.0066
*D*	−0.02702	−0.01935	−0.08942

Note: *B*, *C*, and *D* denote the selected FBG response features obtained from the sensor groups and virtual response combinations used for load identification.

**Table 8:** Validation error metrics for bending moment, shear force, and torsion under Conditions 7 and 8.

Section	Component	n	MAE	RMSE	MaxAE	Bias	Avg. Relative Error/%	Max Relative Error/%	95% CI of Bias	Unit
Section 1	M	20	0.0782	0.1132	0.3537	+0.0119	1.57	6.53	[−0.0421, +0.0660]	kN·m
Section 1	Q	20	0.0507	0.0590	0.1082	−0.0051	0.82	1.64	[−0.0333, +0.0232]	kN
Section 1	T	20	0.0804	0.1066	0.3120	+0.0080	1.15	2.29	[−0.0431, +0.0590]	kN·m
Section 2	M	20	0.0395	0.0500	0.1108	−0.0071	1.15	2.66	[−0.0309, +0.0167]	kN·m
Section 2	Q	20	0.0638	0.0779	0.1338	+0.0119	0.94	2.62	[−0.0250, +0.0489]	kN
Section 2	T	20	0.0738	0.0958	0.2714	+0.0204	1.09	2.09	[−0.0245, +0.0654]	kN·m
Section 3	M	20	0.0322	0.0399	0.0900	−0.0063	1.81	4.43	[−0.0252, +0.0127]	kN·m
Section 3	Q	20	0.0238	0.0283	0.0587	+0.0080	0.82	1.82	[−0.0051, +0.0210]	kN
Section 3	T	20	0.0531	0.0715	0.1590	−0.0185	1.45	4.04	[−0.0517, +0.0146]	kN·m
Overall	M	60	0.0500	0.0751	0.3537	−0.0005	1.51	6.53	[−0.0200, +0.0191]	kN·m
Overall	Q	60	0.0461	0.0587	0.1338	+0.0050	0.86	2.62	[−0.0103, +0.0202]	kN
Overall	T	60	0.0691	0.0925	0.3120	+0.0033	1.23	4.04	[−0.0208, +0.0274]	kN·m

Note: M, Q, and T denote bending moment, shear force, and torsional moment, respectively. MAE, RMSE, MaxAE, and Bias are expressed in the corresponding physical units of each load component. Bias is defined as the mean signed error, namely the predicted value minus the applied reference value. The 95% CI was calculated from the signed validation residuals using Student’s *t*-distribution.

**Table 9:** Rank and condition number of the FBG response-feature matrix used for regression analysis.

Section	Matrix Rank	Condition Number	Evaluation
Section 1	3	3.63	Good conditioning
Section 2	3	2.78	Good conditioning
Section 3	3	19.67	Acceptable conditioning

**Table 10:** Comparison of FBG- and resistance-strain-gauge-based load-identification errors under Conditions 7 and 8.

Load Type	Sensor Type	Maximum Error	Average Error	MAE	RMSE	MaxAE	Bias
M	FBG	6.53%	1.51%	0.0500	0.0751	0.3537	−0.0005
	Strain Gauge	4.61%	1.44%	0.0498	0.0644	0.1888	0.0083
Q	FBG	2.62%	0.86%	0.0461	0.0587	0.1338	0.0050
	Strain Gauge	4.07%	2.74%	0.1452	0.1691	0.3435	0.0033
T	FBG	4.04%	1.23%	0.0691	0.0925	0.3120	0.0033
	Strain Gauge	3.09%	0.88%	0.0489	0.0600	0.1535	0.0047

**Table 11:** Cross-reference summary of the experimental configuration, regression analysis, validation results, and data availability.

Reproducibility Item	Main-Text Location	Detailed Numerical Data	Description
Test article and finite-element model	Section 3.1, Table 1, Figure 1	Table S1 in Supporting Material S1	Specimen parameters, boundary condition, element type, mesh size, node/element counts, and mesh-convergence results.
Loading geometry and section-load calculation	Section 3.3, Table 3, Table 4 and Table 5	Table 3, Table 4 and Table 5	Coordinates of F1–F4, coordinates of Section 1, Section 2 and Section 3, reference torsion axis, sign convention, numerical lever arms, and calculated section loads.
FBG sensor layout	Section 3.2, Figure 3	Table 2 and Figure 3	Coordinates, monitored section, structural region, measurement direction, and feature group of each physical FBG strain sensor.
Instrumentation and bonding procedure	Section 3.4	Table S3 in Supporting Material S1	FBG model, interrogator model, adhesive type, bond thickness, curing protocol, strain-gauge model, bridge/DAQ model, software, sampling rate, and calibration basis.
Temperature compensation and preprocessing	Section 3.4, Figure 8	Tables S4 and S5 in Supporting Material S1	Reference FBG locations, channel-pairing relationships, stable-window definition, Hampel filtering rule, rejected-point statistics, and compensation performance for all sections.
FBG regression model	Section 4.2, Table 7 and Table 8	Table 7 and Table 8	Definition of virtual features B, C, and D, regression coefficients, rank, condition number, and calibration statistics.
FBG validation results	Section 4.3, Figure 6, Table 8	Supporting Material S3	Point-by-point applied loads, regression-predicted loads, absolute errors, and relative errors for Conditions 7 and 8.
Feature ablation analysis	Section 4.3	Table S6 in Supporting Material S1	Leave-one-virtual-feature-out ablation results, including MAE, RMSE, MaxAE, Bias, relative errors, and RMSE increase.
Resistance strain-gauge benchmark	Section 4.4, Table 10	Supporting Material S2	Strain-gauge layout, correspondence with FBG channels, regression formulation, coefficients, calibration–validation split, and point-by-point validation results.
Uncertainty and residual-strain assessment	Section 3.4 and Section 4.4	Tables S2 and S7 in Supporting Material S1	Post-unloading residual-strain statistics, actuator-force uncertainty, wavelength resolution, strain sensitivity, temperature-compensation residuals, repeatability, strain transfer, and regression uncertainty.
Data availability	Data Availability Statement	Supporting Material S3	List of processed datasets provided for reproducing the regression and validation analysis, and statement on unavailable raw time histories if applicable.

## Data Availability

The original contributions presented in this study are included in the article. Further inquiries can be directed to the corresponding author.

## Supplementary Materials

The following supporting information can be downloaded at: https://www.mdpi.com/article/10.3390/s26144650/s1, Table S1: Mesh-sensitivity and convergence results of the finite -element model.; Table S2: Post-unloading residual-strain statistics obtained from the FBG sensors and resistance strain gauges.; Table S3: Instrumentation models, installation procedures, calibration bases, and specified uncertainty limits.; Table S4: Locations and channel-pairing relationships of the strain-free reference FBG sensors.; Table S5: Temperature-compensation and preprocessing statistics for all monitored sections and loading conditions.; Table S6: Leave-one-virtual-feature-out ablation results for the FBG-based load-identification model.; Table S7: Uncertainty sources and propagation paths for the FBG and resistance-strain-gauge load-identification systems. The processed datasets supporting the regression calibration and validation results are provided in the Supplementary Materials. Supporting Material S1 includes mesh-sensitivity results, residual-strain and uncertainty-related tables, instrumentation and bonding information, temperature-compensation statistics, and feature-ablation results. Supporting Material S2 includes the resistance-strain-gauge benchmark, including sensor layout, regression coefficients, calibration–validation split, and point-by-point validation results. Supporting Material S3 includes the point-by-point FBG validation results under Conditions 7 and 8, including section number; loading condition; load level; applied reference bending moment, shear force, and torsional moment; regression-predicted values; absolute errors in physical units; and relative errors. The loading geometry, actuator coordinates, monitored-section coordinates, numerical lever arms, FBG sensor grouping, and regression coefficient matrices are provided in the main text and cross-referenced in Table 11.
