# Supplementary material for: Fixed-Position Quasi-Static Load Calibration and Identification of an Aluminum Wing-Box Test Section Using Surface-Bonded Fiber Bragg Grating Sensors
Source: Sensors (Basel). 2026 Jul 22;26(14):4650. doi: 10.3390/s26144650 (PMC13419079; doi:10.3390/s26144650)
Supplement: Supplementary file 1 [file sensors-26-04650-s001.zip › sensors-4351313 Supporting Material 1.pdf]

Supporting Material 1

Table S1. Mesh-sensitivity and convergence results of the finite-element model.

Mesh	Global/ Local Size	Nodes	Elements	Bending Stress/ MPa	Δ/%	Bending Strain/με	Δ/%	Torsion Stress/MPa	Δ/%	Torsion Strain/με	Δ/%
Coarse	40/20 mm	15,842	14,976	405.8	3.82	5948	3.58	374.6	3.15	5352	4.24
Medium	30/15 mm	27,316	25,988	421.9	1.22	6169	1.39	386.8	1.58	5589	1.38
Fine, adopted	20/10 mm	58,204	55,936	427.1	–	6256	–	393.0	–	5667	–

Table S2. Post-unloading residual-strain statistics obtained from the FBG sensors and resistance strain gauges.

Section	System	Maximum Response /με	Mean Signed Residual/με	Residual SD/με	Maximum Absolute Residual/με	Residual Ratio/%
Section 1	FBG	795.5	+0.3	1.5	3.2	0.40
Section 2	FBG	795.1	+0.4	1.7	3.6	0.45
Section 3	FBG	643.4	+0.5	1.8	3.9	0.61
Section 1	Strain gauge	784.6	+0.2	1.3	2.9	0.37
Section 2	Strain gauge	781.2	+0.2	1.4	3.1	0.40
Section 3	Strain gauge	632.5	+0.3	1.5	3.4	0.54

Table S3. Instrumentation models, installation procedures, calibration bases, and specified uncertainty limits.

**Item**	**Manufacturer/Model**	**Serial/Certificate**	**Main Specification**	**Installation/Acquisition Protocol**	**Calibration Basis**	**Interval**	**Specified Uncertainty/Limit**
FBG strain sensor	Luna os1100	Batch OS1100-01	Single FBG; 10 mm grating; polyimide coating; approx. 1.2 pm/με; Bragg wavelengths selected within 1525–1565 nm	Surface-bonded on the aluminum skins and webs according to the section-wise sensor layout; 100 Hz processed data used for stable-window averaging	Factory batch characterization	Per batch	Sensitivity tolerance: assumed ±1.0%
Temperature-reference FBG	Luna os1100	Same batch as strain FBGs	Same optical properties as strain FBGs	Installed in strain-isolated PTFE sleeves adjacent to each monitored region; paired with measurement FBGs in the same section and structural region	Same batch characterization as strain FBGs	Per batch	Used for temperature compensation; residual SD included in S5
FBG interrogator	Luna HYPERION si155	CAL-SI155-001	Four channels; 1500–1600 nm; 1 pm wavelength accuracy; operated at 100 Hz in this test	LC/APC input connected through an FC/APC adapter; wavelength tracking using Luna ENLIGHT software	Internal NIST-traceable wavelength reference and reference-FBG check	12 months	1 pm wavelength accuracy
FBG adhesive	3M Scotch-Weld DP490	Lot recorded in laboratory log	Two-component structural epoxy	Aluminum surface abraded with 600-grit paper and degreased with acetone; nominal bondline (50 ± 10) μm; cured for 10 h at (20 ± 2) °C under contact pressure; stabilized for 24 h before testing	Manufacturer curing specification; thickness controlled by spacer	N/A	Bondline uncertainty included in strain-transfer assessment
Resistance strain gauge	HBK 1-LY13-10/120	Batch LY13-01	10 mm grid; 120 Ω; gauge factor 2.08;	Installed adjacent to the corresponding FBG regions; same stable-	Batch-specific gauge-factor sheet	Per batch	Gauge factor: 2.08 ± 1.0%

Item	Manufacturer/Model	Serial/Certificate	Main Specification	Installation/Acquisition Protocol	Calibration Basis	Interval	Specified Uncertainty/Limit
			temperature response matched to aluminum	window averaging procedure as FBG data			
Strain-gauge adhesive	HBK 1-CA80	Lot recorded in laboratory log	Single-component cyanoacrylate adhesive	Thin bondline, nominally <10 µm; thumb pressure for 1 min at 20 °C; relative humidity 40–70%; installed according to manufacturer instructions	Manufacturer installation instructions	N/A	Bondline effect considered in strain-gauge benchmark uncertainty
Bridge conditioner and DAQ	HBK QuantumX MX1615B	CAL-MX1615-001	Sixteen channels; 24-bit ADC; internal 120/350 Ω quarter-bridge completion; up to 20 kS/s; operated at 100 Hz in this test	Three-wire quarter-bridge connection; bridge balancing, filtering, and strain conversion using HBK catman AP software	Factory electrical calibration	12 months	As stated in certificate
Actuator F1	Instron Hydropuls PL, 20 kN configuration	FORCE-F1	20 kN working range	Spherical rod-end joint; downward force defined as positive	Traceable force calibration	12 months	±0.5% FS, equivalent to ±0.10 kN
Actuator F2	Instron Hydropuls PL, 20 kN configuration	FORCE-F2	20 kN working range	Spherical rod-end joint; downward force defined as positive	Traceable force calibration	12 months	±0.5% FS, equivalent to ±0.10 kN
Actuator F3	Instron Hydropuls PL, 20 kN configuration	FORCE-F3	20 kN working range	Spherical rod-end joint; downward force defined as positive	Traceable force calibration	12 months	±0.5% FS, equivalent to ±0.10 kN
Actuator F4	Instron Hydropuls PL, 20 kN configuration	FORCE-F4	20 kN working range	Spherical rod-end joint; downward force defined as positive	Traceable force calibration	12 months	±0.5% FS, equivalent to ±0.10 kN
FBG acquisition software	Luna ENLIGHT	N/A	Peak tracking and wavelength-to-	Common 100 Hz sampling and time stamps; stable-window	Interrogator internal reference	N/A	Included through interrogator wavelength

Item	Manufacturer/Model	Serial/Certificate	Main Specification	Installation/Acquisition Protocol	Calibration Basis	Interval	Specified Uncertainty/Limit
			strain conversion	averaging after temperature compensation			accuracy and repeatability
Strain-gauge acquisition software	HBK catman AP	N/A	Bridge balancing, filtering, and strain conversion	100 Hz sampling; same stable-window averaging as FBG data	MX1615B calibration	N/A	Included through bridge/DAQ and gauge-factor uncertainty

FBG Arrangement and Compensation

Six strain-free reference FBG sensors were used, with two reference sensors assigned to each monitored section. The skin reference and web reference for each section were placed in loose PTFE sleeves immediately adjacent to the corresponding aluminum surface. This configuration maintained local thermal coupling while minimizing the transfer of mechanical strain to the reference FBGs.

Table S4. Locations and channel-pairing relationships of the strain-free reference FBG sensors.

Section	Reference FBG	Location	Paired Measurement Channels
Section 1 (x = 157 mm)	R_{S1}	Skin region, strain-isolated	3 upper-skin + 3 lower-skin FBGs
Section 1	R_{W1}	Web region, strain-isolated	2 front-web + 2 rear-web FBGs
Section 2 (x = 457 mm)	R_{S2}	Skin region, strain-isolated	3 upper-skin + 3 lower-skin FBGs
Section 2	R_{W2}	Web region, strain-isolated	2 front-web + 2 rear-web FBGs
Section 3 (x = 757 mm)	R_{S3}	Skin region, strain-isolated	3 upper-skin + 3 lower-skin FBGs
Section 3	R_{W3}	Web region, strain-isolated	2 front-web + 2 rear-web FBGs

Note: R_{Si} and R_{Wi} denote the skin-region and web-region reference FBGs in Section *i,* respectively. The arrangement resolves spanwise differences and skin-versus-web temperature differences but does not independently resolve smaller gradients within an individual sensor group; the remaining influence is represented by the residual statistics in Table S5.

Stable-Window Selection and Abnormal-Point Removal

1. Each load level was held for 30 s and sampled at 100 Hz, producing 3000 samples per channel.
2. The first 20 s were treated as the loading and stabilization interval and were excluded from averaging. The final 10 s (1000 samples) defined the stable window.
3. A sample was retained only when the deviations of all four actuator forces from their respective targets were within +/-0.5% of the 20 kN full scale (+/-0.10 kN). This absolute full-scale criterion also applies when an actuator target is zero.
4. After force-stability screening, a centered 101-point Hampel filter (approximately 1 s at 100 Hz) was applied independently to each FBG channel.
5. Points identified as abnormal were removed without interpolation. If more than 5% of the samples in a stable window were rejected, the holding segment was discarded and the test was repeated.
6. Temperature compensation was applied to the retained samples before calculating the stable-window mean. One mean was obtained from each repeated test; the average of the three repeat means was used as the regression input, and their standard deviation was retained for repeatability analysis.

$$\left|x_t - median(x)\right| > 3\times 1.4826 \times MAD(x) \tag{S1}$$

Equation S1 defines the Hampel rejection threshold. The median and median absolute deviation (MAD) were calculated within the local 101-point window.

Cross-Section and Cross-Condition Assessment

Temperature-compensation performance was evaluated over 240 holding segments per section, comprising eight loading conditions, ten non-zero load levels, and three repeated tests (8 × 10 × 3). The statistics therefore cover all three monitored sections and all prescribed calibration and validation conditions, rather than only the representative Section 1, Condition 2 example shown in the manuscript.

Table S5. Temperature-compensation and preprocessing statistics for all monitored sections and loading conditions.

Section	Holding Segments, n	Max Raw Thermal Component (Microstrain)	Mean Absolute Residual (Microstrain)	Max Residual (Microstrain)	Raw Repeatability SD (Microstrain)	Compensated SD (Microstrain)	Rejected Points (%)
Section 1	240	18.4	0.8	2.6	4.9	2.4	0.21
Section 2	240	16.7	0.9	2.9	5.2	2.7	0.25
Section 3	240	20.9	1.1	3.4	5.8	3.2	0.34

Note: The maximum raw thermal component is the largest equivalent-strain correction obtained from the local reference FBG. Residual values denote the remaining section-wise apparent-strain component after local compensation over the retained stable-window data. Raw and compensated repeatability SD values are the section-wise means of the standard deviations of the three repeat means evaluated over 80 condition-level combinations. Rejected points are Hampel-identified samples after force-stability screening.

Across Sections 1—3, compensation reduced the repeatability SD from 4.9-5.8 microstrain to 2.4-3.2 microstrain, corresponding to reductions of approximately 44.8-51.0%. The maximum residual was 3.4 microstrain, and the rejected-point proportion remained below 0.35% in every section.

Table S6. Leave-one-virtual-feature-out ablation results for the FBG-based load-identification model.

Configuration	Component	MAE	RMSE	MaxAE	Bias	Avg. RE/%	Max RE/%	RMSE increase/%
Baseline B+C+D	M	0.0500	0.0751	0.3537	-0.0005	1.51	6.53	0
Baseline B+C+D	Q	0.0461	0.0587	0.1338	+0.0050	0.86	2.62	0
Baseline B+C+D	T	0.0691	0.0925	0.3120	+0.0033	1.23	4.04	0
Without B	M	0.1416	0.1877	0.6092	-0.1416	3.99	9.76	150
Without B	Q	0.0477	0.0705	0.1945	-0.0251	0.88	2.50	20
Without B	T	0.0967	0.1387	0.3872	-0.0789	1.74	4.73	50
Without C	M	0.0773	0.1126	0.5012	+0.0680	2.31	5.65	50
Without C	Q	0.1113	0.1469	0.4008	-0.1108	2.00	4.04	150
Without C	T	0.1243	0.1665	0.5178	+0.1166	2.30	5.06	80
Without D	M	0.1055	0.1351	0.3623	-0.0983	3.16	9.19	80
Without D	Q	0.0763	0.1057	0.3195	-0.0713	1.38	2.96	80
Without D	T	0.2256	0.2774	0.6712	-0.2256	3.94	7.70	200

Table S7. Uncertainty sources and propagation paths for the FBG and resistance-strain-gauge load-identification systems.

Source	Limit/Statistic	Distribution	Divisor	Standard Uncertainty	Propagation/Usage
Actuator force	±0.10 kN	Rectangular	√3	0.0577 kN per actuator	Propagated through section-load equations
Loading-point coordinate	±1.0 mm	Rectangular	√3	0.577 mm	Propagated through moment and torsion arms
Interrogator wavelength	±1 pm	Rectangular	√3	0.577 pm	Converted to equivalent FBG strain uncertainty
Equivalent wavelength strain	0.577 pm / 1.2 pm/με	Propagated	—	0.481 με	Contribution to FBG feature uncertainty
FBG sensitivity	±1.0%	Rectangular	√3	0.577%	Multiplicative contribution to FBG strain features
Temperature compensation	Residual SD = 1.5 με	Normal	1	1.5 με	Contribution to FBG feature uncertainty
Repeatability of mean	SD = 0.81–4.13 με, n = 3	Normal	√3	0.47–2.38 με	Contribution to stable-window mean uncertainty
FBG strain transfer	±1.0%	Rectangular	√3	0.577%	Multiplicative contribution associated with surface bonding
Bond thickness	(50 ± 10) μm	Rectangular	√3	5.77 μm	Included through strain-transfer assessment
Gauge factor	±1.0%	Rectangular	√3	0.577%	Contribution to resistance-strain-gauge benchmark
Regression residual, M	RMSE = 0.0751 kN·m	Empirical normal	1	0.0751 kN·m	Empirical prediction uncertainty for bending moment
Regression residual, Q	RMSE = 0.0587 kN	Empirical normal	1	0.0587 kN	Empirical prediction uncertainty for shear force
Regression residual, T	RMSE = 0.0925 kN·m	Empirical normal	1	0.0925 kN·m	Empirical prediction uncertainty for torsional moment
